# A Quantitative Human Red Blood Cell Agglutination Assay for Characterisation of Galectin Inhibitors

**DOI:** 10.3390/ijms25126756

**Published:** 2024-06-19

**Authors:** Rhianna Gasson, James A. Roper, Robert J. Slack

**Affiliations:** Galecto Biotech AB, Stevenage Bioscience Catalyst, Stevenage SG1 2FX, UK

**Keywords:** red blood cell agglutination, galectin-3, galectin-1, assay optimisation

## Abstract

Galectins are a family of beta-galactoside-binding proteins that are characterised by their carbohydrate recognition domain (CRD) and include galectin-1 and galectin-3. These galectins have been implicated in numerous diseases due to their pleiotropic nature, including cancer and fibrosis, with therapeutic inhibitors being clinically developed to block the CRD. One of the early methods developed to characterise these galectins was the hemagglutination of red blood cells. Although it is insightful, this approach has been hampered by a lack of sensitivity and accurate quantification of the agglutination observed. In this study, we aimed to validate a more precise and quantitative method to enable the further investigation of differences between galectins in respect to agglutination induction in different blood groups, as well as the characterisation of small molecule inhibitors. Quantification of hemagglutination was shown to be optimal using U-bottom plates imaged and analysed with FIJI ImageJ rather than flat-bottom plates read for absorbance on an optical density plate reader. Galectin-3-induced red blood cell agglutination efficacy increased significantly from blood group O to A to B. However, for both the galectin-1 monomer and concatemer, a more comparable effect was observed between blood group B and O, but with more potent effects than in blood group A. Inhibition assays for both galectin-3 and galectin-1 induced-hemagglutination were able to demonstrate clear concentration responses and expected selectivity profiles for a set of small-molecule glycomimetics, confirming the historical profiles obtained in biochemical binding and functional cellular assays.

## 1. Introduction

Galectins are a family of beta-galactoside-binding proteins that are characterised by their carbohydrate recognition domain (CRD) [1]. The family includes galectin-3, the only member of the chimeric sub-family that is able to undergo oligomerisation to form lattice structures, which play a pleiotropic role in the development of fibrosis and cancer [2,3,4]. This occurs via the binding of the CRDs to glycans on cell surface receptors, disrupting or exacerbating a number of different mechanisms [5]. In addition, galectin-1 is a member of the prototypical galectin sub-family that can also bind glycans, but as a monomer or dimer, resulting in the modulation of the immune response and pro-oncogenic pathways [6,7]. Elevation of galectin-1 and galectin-3 levels in the target organs and blood are observed across the indications which they are implicated in [2,6]. This suggests that the systemic levels of these galectins are influenced by the upregulation in the diseased organ, and therefore this could be exploited as a surrogate biomarker of prognosis and/or therapeutic intervention. Therefore, any blood donor differences in respect to measuring free levels of galectin could be impactful when comparing healthy and patient populations or treated and untreated patient cohorts.

**Figure 1:**
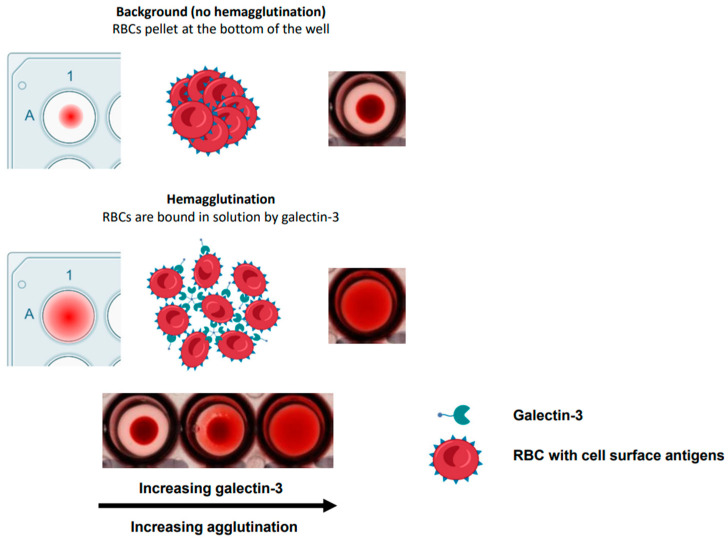
Schematic depicting galectin-3-induced hemagglutination in human red blood cells. Red blood cell (RBC).

As a result of their pro-fibrotic and pro-tumorigenic effects, galectin-3 and galectin-1 have been investigated as potential therapeutic targets, with small- and large-molecule inhibitors in development [6]. The generation of simple and robust functional in vitro assays for measuring the effects of galectins, and inhibitors thereof, can be a challenge. However, one of the methods that has been used is galectin-induced hemagglutination of red blood cells (RBCs) [8,9,10], as summarised in Figure 1. These assays offer a simple, fast, and relatively high throughput approach to demonstrating functional activities of different protein batches and constructs of galectins across species in a binary fashion, although they have yet to be used for showing the efficacy and selectivity of glycomimetic inhibitors. Historically, the main drawback of using RBC hemagglutination has been the lack of quantitative and accurate readouts, where analysis has been confined to visual observations [8,9,10].

The aim of this study was to develop quantitative RBC agglutination assays for galectin-1 and -3, whereby sensitive readouts would allow for more insight into the effects of these proteins on different blood groups, in addition to allowing for the analysis of concentration response effects of small molecule glycomimetic inhibitors.

## 2. Results

### 2.1. Optimisation of Hemagglutination Assay

**Figure 2:**
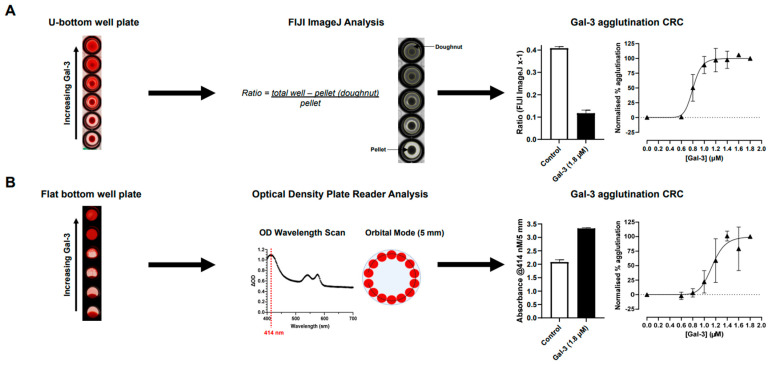
Optimization of the quantification of galectin-3-induced hemagglutination in (**A**) U-bottom-well plates using FIJI ImageJ analysis or (**B**) flat-bottom-well plates using an optical density plate reader. Raw data from each analysis are from 4 blood group A donors, with mean ± SD. Data shown in concentration–response curves (black triangles) are from one representative donor, with *n* = 3, technical replicates displayed as mean ± SD. CRC, concentration–response curve; Gal-3, galectin-3.

In order to optimise the quantification of galectin-induced RBC agglutination, different plates and analysis methods were applied. As part of the initial assay development, the concentration of blood resuspended in Alsever’s solution was optimised, with 3.75% shown to be the most robust, in respect to the control RBC pellet size not being too large that the whole well was occluded, an observable smooth gradient effect across the concentrations of galectin-3, and full induction of RBC agglutination at the highest concentration of galectin-3 (Figure S1). In addition, with 3.75% Alsever’s solution, the hemagglutination was shown to be more stable over time to enable multiple experiments to be completed from a single donor (data shown). Using the optimised 3.75% Alsever’s solution concentration, U-bottom and flat-bottom plates were used to compare quantification techniques using either FIJI ImageJ or plate reader analysis, respectively (Figure 2). Over four donors of blood group A, a signal-to-background ratio of 3.5 was observed for U-bottom/FIJI ImageJ analysis, compared with 1.6 for the flat-bottom/plate reader analysis. In addition, the variability between technical replicates and the quality of *EC*_50_ fit for galectin-3 within a single donor was better for FIJI ImageJ (r^2^ = 0.94) compared with an optical density plate reader analysis (r^2^ = 0.83) (Figure 2).

### 2.2. Comparison of Galectin-3-Induced Hemagglutination across Blood Groups A, B, and O

**Figure 3:**
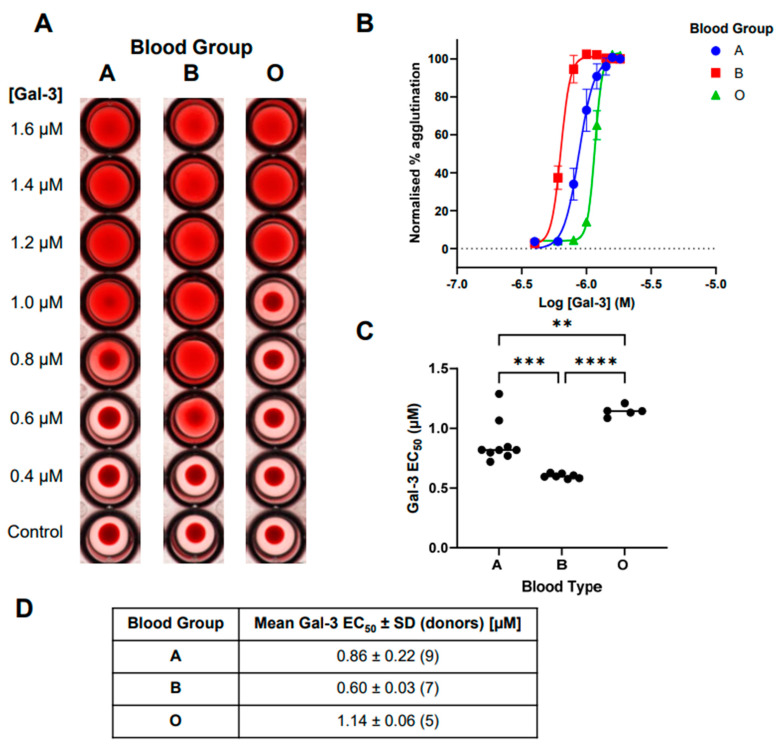
Galectin-3-induced red blood cell agglutination across A, B, and O blood groups. (**A**) Example visual representation of galectin-3-induced hemagglutination across blood groups in single donors. (**B**) Concentration–response curves of galectin-3-induced hemagglutination across blood groups for all donors (mean ± SEM), with *EC*_50_ values shown in (**C**,**D**). Statistical comparisons completed with ANOVA (Tukey post-test), with ** *p* < 0.01, *** *p* < 0.001, and **** *p* < 0.0001.

When comparing concentration curves, a clear difference between galectin-3-induced hemagglutination across blood groups A, B, and O was visually observed (Figure 3A). This was further confirmed across multiple donors when data was analysed in FIJI ImageJ (Figure 3B). The resulting *EC*_50_ values showed a significant difference between all blood groups tested (Figure 3C,D), with the order of increasing efficacy from O though A and then B being demonstrated.

### 2.3. Comparison of Galectin-1-Induced Hemagglutination across Blood Groups A, B, and O

**Figure 4:**
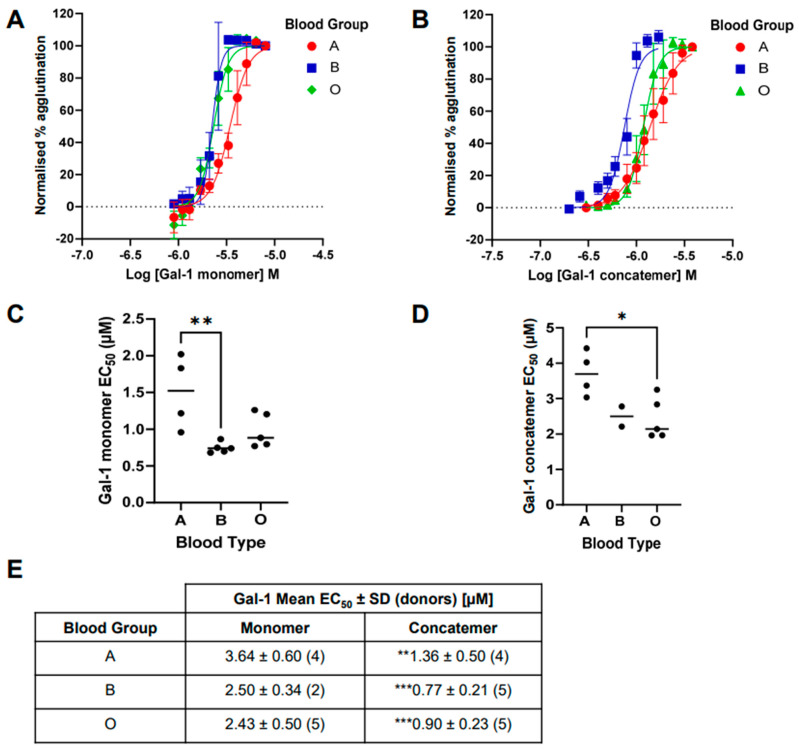
Galectin-1-induced red blood cell agglutination across A, B, and O blood groups. (**A**) Concentration-response curves of galectin-1 monomer-induced hemagglutination across blood groups for all donors (mean ± SEM), with *EC*_50_ values shown in (**C**,**E**). (**B**) Concentration–response curves of galectin-1 concatemer-induced hemagglutination across blood groups for all donors (mean ± SEM), with *EC*_50_ values shown in (**D**,**E**). Statistical comparisons between blood groups completed with ANOVA (Tukey post-test), with * *p* < 0.05 and ** *p* < 0.01. Statistical comparisons between galectin-1 monomer and concatemer within blood groups, completed with a Student’s *t*-test, with ** *p* < 0.01 and *** *p* < 0.001.

The variability of galectin-1 induced hemagglutination across blood groups was greater with both the monomer and concatemer forms (Figure 4) compared to that observed with galectin-3 (Figure 3). This made the comparison between the effects in different blood groups more difficult. However, for the galectin-1 monomer, a more potent *EC*_50_ for RBC agglutination was observed in blood group B and O compared with A, although this was only significant between A and B. For the galectin-1 concatemer, a similar trend was observed, with a more potent *EC*_50_ for RBC agglutination observed in blood group B and O compared with A, although for the concatemer, it was only significant between A and O. The concatemer of galectin-1 demonstrated a more potent hemagglutination effect than that observed for the monomer within in each of the blood groups tested. The concatemer of galectin-1 had comparable *EC*_50_ values to galectin-3 for each blood group, whilst the monomer was weaker.

### 2.4. Inhibition of Galectin-Induced Hemagglutination with Small-Molecule Glycomimetic and Allosteric Inhibitors

**Figure 5:**
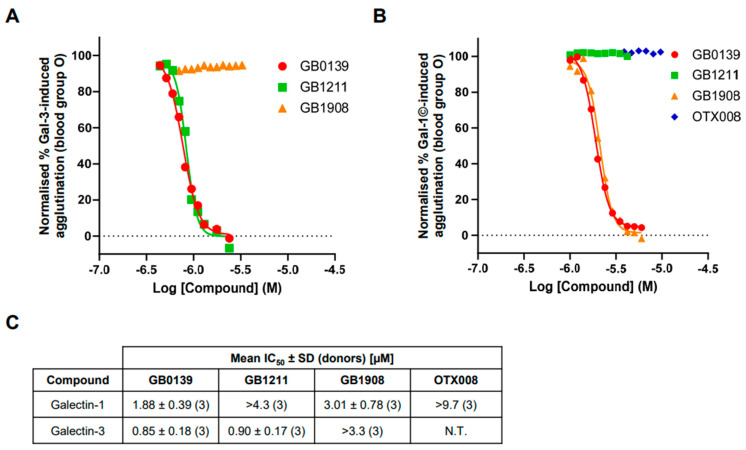
Inhibition of galectin-induced hemagglutination by small-molecule inhibitors. (**A**) Inhibition of galectin-3-induced hemagglutination in blood group O, with representative concentration-response curves for GB0139, GB1211, and GB1908 at a fixed concentration of galectin-3 (1.6 µM). (**B**) Inhibition of galectin-1 concatemer-induced hemagglutination in blood group O, with representative concentration-response curves for GB0139, GB1211, GB1908, and OTX008 at a fixed concentration of galectin-1 (1.2 µM). Where measurable, *IC*_50_ values for inhibitors against galectin-1 and galectin-3 are shown in (**C**). Gal-1©, galectin-1 concatemer; N.T., not tested.

To investigate the selective inhibition of galectin-1 and galectin-3 inhibitor and determine IC_50_ values, hemagglutination at fixed concentrations of galectins was investigated in blood group O (Figure 5). The galectin-3 inhibitors GB0139 and GB1211 demonstrated a concentration-dependent inhibition of galectin-3-induced RBC agglutination with comparable *IC*_50_ values (Figure 5A,C). The selective galectin-1 inhibitor GB1908 showed no inhibition across the concentrations tested up to 3.3 µM. The dual galectin-1 and -3 inhibitor GB0139 and selective galectin-1 inhibitor GB1908 both demonstrated a concentration-dependent inhibition of galectin-1 concatemer-induced RBC agglutination with comparable *IC*_50_ values (Figure 5B,C). The selective galectin-3 inhibitor GB1211 showed no inhibition across the concentrations tested up to 4.3 µM. Interestingly, the allosteric galectin-1 inhibitor OTX008 also showed no inhibition across the concentrations tested up to 9.7 µM.

## 3. Discussion

Galectins are rapidly becoming of interest as drug targets due to their negative pleotropic role in many diseases, with a number of companies now having small- and large-molecule inhibitors in clinical development [1,2,3,6]. One of the routine academic assays that has been used for measuring the effects of galectins on biological function, that has included differences between protein sequences and blood groups, has been hemagglutination of RBCs [8,9,10]. Although useful for demonstrating the effects of these variables, the results have usually been binary in respect to response. Therefore, in this study, our aim was to further develop these assays into a format that was able to be more quantitative and enable the generation of robust concentration response effects not only for galectins, but also for inhibitors thereof. 

The readout for RBC agglutination has relied on visual inspection of the reaction vessel that only enables a crude estimate of the concentration effect of the stimulant or inhibitor. Therefore, the quantification of hemagglutination was investigated using either an absorbance plate reader or plate imaging combined with a FIJI ImageJ analysis to improve the sensitivity of the readout. Both of these approaches came with their strengths and weaknesses. The plate reader’s output required a longer incubation period to enable a robust absorbance measurement, but with the benefit of no manual data analysis. This was compared with a shorter incubation period with the imaged plate format, but had a more manual and labour-intensive data analysis. In comparison, these pros and cons equated to a similar length of time for each assay format to be completed, which meant that the method with the optimal performance in respect to data quality would be selected to be taken forward for further experiments. This was demonstrated to be the method of imaging the plate and applying a ratio analysis with FIJI ImageJ that allowed for a more robust differentiation between the non-agglutinated cell pellet and agglutinated RBCs in the well of a U-bottom plate. The requirement for a flat bottom well to enable an absorbance readout on a plate reader resulted in a more variable output, as plates were required to be incubated at an angle to allow for the differentiation between agglutinated and non-agglutinated RBCs. The aid of a natural basin for cell pellets in the U-bottom well likely resulted in providing the more robust analysis output. 

With the optimal quantification method identified, a comparison of galectin-1 and -3-induced hemagglutination was completed against blood groups A, B, and O to allow for the comparison with historical analysis using the visual binary readouts in the literature. For galectin-3, hemagglutination across blood groups A, B, and O significantly differed with increasing efficacy from O though A and then B. This confirmed the historical observations by demonstrating a difference between A and B compared with O [8]. However, the more sensitive analysis in our method allowed for a significant difference to also be observed between blood groups A and B for the first time. For galectin-1, the difference observed by Shu and co-workers [10] between the monomer and concatemer of this galectin was repeated, and was now shown to be significant, with a 2.7–3.3-fold increase in *EC*_50_ values across the three blood groups tested. Although the galectin-1 effects were overall more variable, differences could be observed between blood groups that had not been observed below. In previous studies, it has been suggested that there were no difference in the ability of a monomer of galectin-1 to induce RBC agglutination across blood groups A, B, and O [8]. Interestingly, we have shown that there is a difference between blood groups B and O compared with A, with a weaker effect in the latter. This observation suggests that there is a preference of galectin-1 for blood group cell surface glycans.

Full inhibition curves for small-molecule glycomimetic inhibitors of both galectin-1 and -3 were generated for the first time using this assay method, which enabled *IC*_50_ values to be generated and compared. In an assay where a galectin is exogenously added, the *IC*_50_ values for an inhibitor will be directly related to the galectin concentration [11]. Therefore, it would be expected that, in the assays completed in this study, that *IC*_50_ values would be the same for all compounds, even if their affinity for the galectin differed, as was observed. This assumes that the concentration of galectin added (µM) is much higher than the compound affinities, as was the case here, as all small-molecule glycomimetics have been demonstrated to have nM affinities [12,13,14]. The expected selectivity profile of the inhibitors tested was observed with the dual galectin-1 and -3 inhibitor GB0139 attenuating the effects of both galectins tested, and the selective galectin-3 inhibitor GB1211 and selective galectin-1 inhibitor GB1908 only inhibiting their respective lectin binding partner. Interestingly, the proposed allosteric inhibitor of galectin-1, OTX008, showed no inhibition in the galectin-1 hemagglutination assay. This would either suggest that the concentrations tested were not high enough, as it has been shown to be weak allosteric binder [15], or that this binding modality did not influence the galectin-1 CRD engagement and the induction of RBC agglutination in this assay.

Although galectin-induced hemagglutination is not a physiologically relevant process per se in respect to a disease outcome, an inhibitor of these lectins would be designed to tackle the biological consequence of different galectin–blood group epitope relationships, which could impact the biology of these galectins as well as their use as clinical biomarkers. Recent studies have demonstrated that, in the context of heart disease, blood group differences are observed when investigating galectin-3, where modulation of its biology and interactions with other proteins is different for patients with different blood groups [16]. With a difference shown here for the first time with galectin-1, a similar effect could be hypothesised in diseases where this lectin is driving progression, and, therefore, future biomarker data assessing either disease prognosis or therapeutic intervention should take an individual’s blood group into consideration as part of data analysis.

To increase throughput, speed, and thus enable a more screening application for the method we describe here, the assay could be further improved by applying an automated analysis in FIJI ImageJ and by potentially investigating miniaturisation into a 384-well plate format. In summary, we have demonstrated in this study that the basic, binary galectin-induced hemagglutination assays can be optimised to be more robust and quantitative, enabling a more detailed pharmacological characterisation of galectin and galectin inhibitor effects in a functional cell system.

## 4. Materials and Methods

### 4.1. Materials

Unless otherwise stated, all small molecule galectin inhibitors were synthesized by the Medicinal Chemistry Department at Galecto Biotech AB (Gothenburg, Sweden). The following galectin inhibitors were tested: GB0139 (dual human galectin-1/3 inhibitor) [12,17,18], GB1211 (human galectin-3 inhibitor) [13,19], GB1908 (human galectin-1 inhibitor) [14], and OTX008 (allosteric galectin-1 inhibitor, also known as Calixarene 0118, was purchased from MedChemExpress (Monmouth Junction, NJ, USA)) [15]. All compound stocks were made up of 100% dimethyl sulfoxide (DMSO) at 30 mM, except for OTX008, which, due to poor solubility in DMSO, was made up to 10 mM in 100% ethanol, and then was tested at a final DMSO or ethanol final assay concentration of 0.1%. Galectin-1 monomer and concatemer were generated as previously described [10,20]. Galectin-3 monomer was generated as previously described [21]. All reagents and plasticware were purchased from ThermoFisher Scientific (Waltham, MA, USA), unless otherwise stated.

### 4.2. Red Blood Cell Preparation

Whole blood from groups A, B, and O were obtained from NHS Blood and Transplant, with approval granted by the South Central (Hampshire B) Research Ethics Committee (REC) under REC reference 21/SC/0238. Whole blood was diluted to 20% in phosphate-buffered saline (PBS), washed 4× in PBS (each centrifugation step at 600× *g* for 20 min at room temperature), and then was resuspended between 2.5% and 10% in Alsever’s solution. Red blood cell (RBC) counts were completed using a Neubauer cell counter before washing, and then again prior to final resuspension in Alsever’s solution, to negate the loss of RBCs during the wash steps and resuspension.

### 4.3. Hemagglutination Assay

#### 4.3.1. Optimisation of Hemagglutination Quantification

For measuring galectin-induced hemagglutination, 50 µL galectin (at varying concentrations) and 50 µL of 3.75% whole blood in Alsever’s solution were added to either a flat (flat clear-bottom black polystyrene TC-treated 96-well microplate (Corning, Glendale, AZ, USA)) or round (U)-bottom (Nunc™ round-bottom clear polystyrene 96-well microplate) plate. Then, solutions were mixed gently using a pipette. Plates were incubated on a plate shaker for 30 min, followed by 1 h on the bench, both at room temperature. Flat-bottom plates were then tilted at a 45° angle and incubated for 18 h prior to reading absorbance at 414 nm (orbital scan, with the reader measuring each well along a defined orbit (5 mm) calculating an average from the data collected) on a CLARIOstar^®^ multi-mode plate reader (BMG Labtech, Ortenberg, Germany) (Figure 2). U-bottom plates remained flat on the bench for 3 h prior to imaging on an ImageQuant™ 800 (Cytiva, Marlborough, MA, USA). Images from U-bottom plates were then analysed using FIJI ImageJ software (version 1.53t) to determine the relative intensity of the RBC pellet and non-RBC pellet (defined as doughnut area) well areas, which were then ratioed (see Figure 2). The total well and the RBC pellet areas were measured using FIJI ImageJ, and a mean grey value of each measurement area was generated. The total well measurement was the area controlled by the software, where the same measurement area was used for all wells on a per-column basis. This was selected because the angle of the plate image was shown to affect the shape of the well, meaning that one particular shape or area may not have fit all columns, and could have resulted in an unrepresentative well area being captured. For measuring RBC pellets, the pellet area was drawn for each well individually, and in cases where there were no pellets, e.g., high galectin, a vehicle control well from the same column was used to define the pellet area in that well. The doughnut area was calculated by subtracting the RBC pellet area from the total well measurement in each well, giving the mean grey value of the well with the pellet excluded. The ratio of the doughnut to the pellet was then determined by dividing the doughnut area by the RBC pellet area for each well.

*EC*_50_ values for galectin-3, galectin-1, and galectin-1 concatemer were determined across blood groups using optimised conditions. For the determination of galectin *EC*_50_ values from both analysis methods, raw hemagglutination data were normalized to vehicle control (0% agglutination) and the maximum concentration of galectin added (100% agglutination). Concentration–response curve data were fitted to a non-linear regression curve (four parameter, variable slope) to obtain the *EC*_50_ values.

#### 4.3.2. Determining Galectin Inhibitor Hemagglutination IC_50_ Values

To determine the *IC*_50_ values for galectin inhibitors in the hemagglutination assay, inhibitors at a range of concentrations were tested at fixed concentrations of galectin using the general protocol described above and quantification with the FIJI Image J analysis method. Blood group O was selected based on the availability of and most robust donor-to-donor galectin *EC*_50_ values to then investigate the profile of galectin-1 and galectin-3 small-molecule inhibitors. For the optimised signal window, the fixed galectin concentrations were selected based on the *EC*_50_ value determined for each galectin against blood group O. For galectin-3, a standardised concentration of 1.6 µM for blood group O was selected, whilst for galectin-1, concatemer 1.2 µM was selected. For the determination of galectin inhibitor *IC*_50_ values, hemagglutination data were normalized to vehicle + galectin control (100% agglutination) and the maximum concentration of galectin inhibitor added (0% agglutination). Concentration–response curve data were fitted to a non-linear regression curve (four parameter, variable slope) to obtain *IC*_50_ values.

### 4.4. Data Analysis

Statistical analyses were completed using Prism 10.2 (GraphPad Software, San Diego, CA, USA). A Student’s *t*-test was used to compare two datasets, and one-way analysis of variance (ANOVA) was used for the comparison of more than two datasets, with an appropriate post-test completed where significance was observed. Each condition tested within each individual experiment (donor) was the mean of three technical replicates.

## Data Availability

The data presented in this study are available on request from the corresponding author.

## Supplementary Materials

The following supporting information can be downloaded at: https://www.mdpi.com/article/10.3390/ijms25126756/s1.
