# Supplementary material for: A Quantitative Human Red Blood Cell Agglutination Assay for Characterisation of Galectin Inhibitors
Source: Int J Mol Sci. 2024 Jun 19;25(12):6756. doi: 10.3390/ijms25126756 (PMC11204262; doi:10.3390/ijms25126756)
Supplement: Supplementary file 1 [file ijms-25-06756-s001.zip › ijms-3015385-supplementary.pdf]

Supplementary Figure

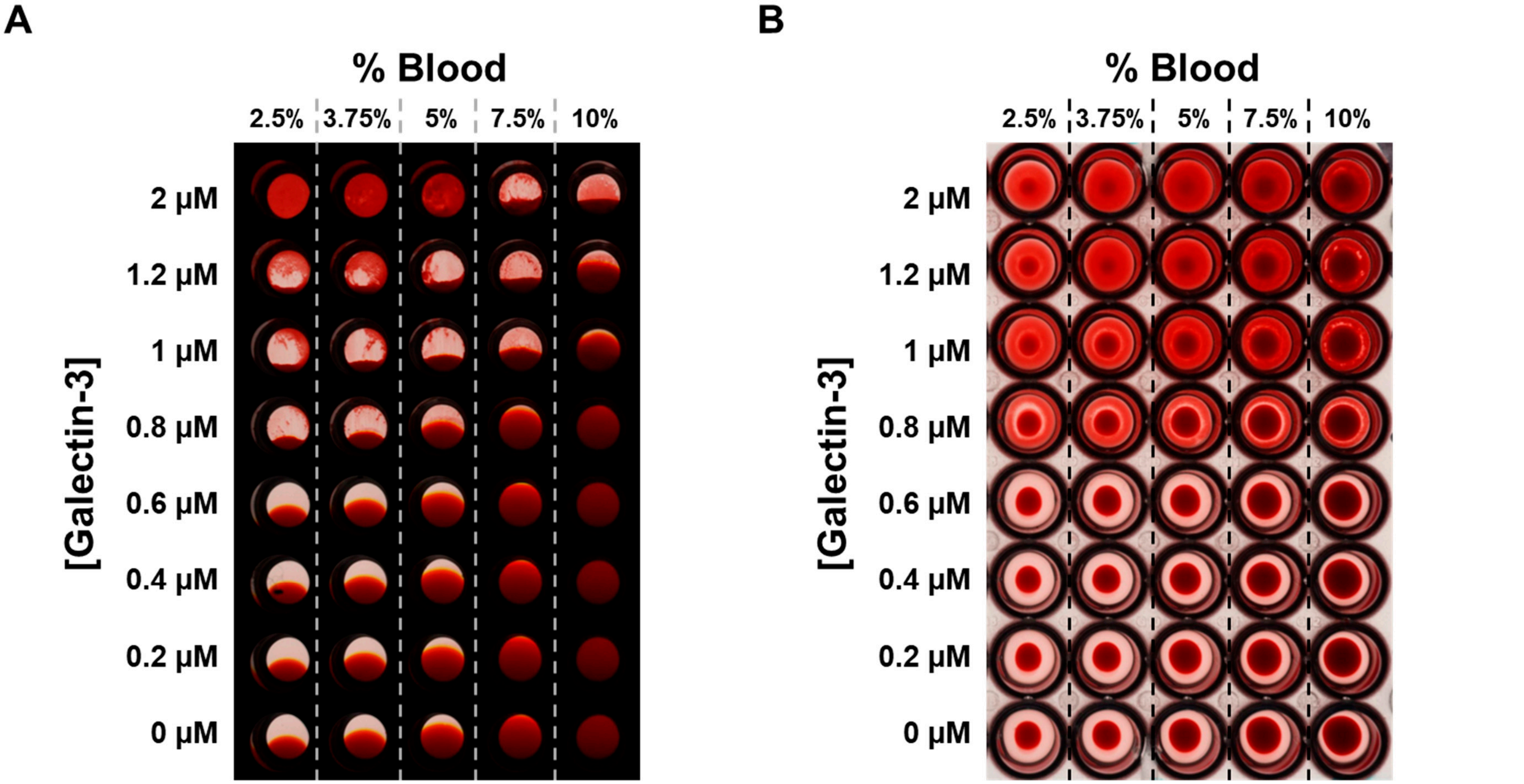

Supplementary Figure S1. Optimization of the % blood used for galectin-induced hemagglutination in (**A**) flat bottom and (**B**) U-bottom well plates.
